# Phenotypic variability of the kyphoscoliotic type of Ehlers-Danlos syndrome (EDS VIA): clinical, molecular and biochemical delineation

**DOI:** 10.1186/1750-1172-6-46

**Published:** 2011-06-23

**Authors:** Marianne Rohrbach, Anthony Vandersteen, Uluç Yiş, Gul Serdaroglu, Esra Ataman, Maya Chopra, Sixto Garcia, Kristi Jones, Ariana Kariminejad, Marius Kraenzlin, Carlo Marcelis, Matthias Baumgartner, Cecilia Giunta

**Affiliations:** 1 Division of Metabolism, University Children's Hospital and Children's Research Centre, (CRC) Zurich, Switzerland; 2 Connective Tissue Unit, University Children's Hospital and Children's Research Centre, (CRC) Zurich, Switzerland; 3 NW Thames Regional Genetics Unit, Kennedy Galton Centre, North West London Hospitals NHS Trust, Harrow, Middlesex and Ehlers-Danlos Syndrome National Diagnostic Service, UK; 4 Gaziantep Children's Hospital, Division of Child Neurology, Gaziantep, Turkey; 5 Ege University Medical School, Department of Pediatrics, Division of Child Neurology Izmir, Turkey; 6 Ege University Medical School, Department of Medical Genetics, Izmir, Turkey; 7 Children's Hospital at Westmead, Department of Clinical Genetics and University of Sydney, Discipline of Paediatrics and Child Health, Sydney, Australia; 8 Unidad de Genètica Clínica i Citogenética, Hospital Materno Infantil Vall d'Hebron, Barcelona, Spain; 9 Kariminejad-Najmabadi Pathology & Genetics Center, Tehran, Iran; 10 Division of Endocrinology, Diabetes, and Clinical Nutrition, University Hospital, Basel, Switzerland; 11 Department of Human Genetics, St Radboud University Medical Centre Nijmegen, Nijmegen, the Netherlands

## Abstract

**Background:**

The kyphoscoliotic type of Ehlers-Danlos syndrome (EDS VIA) (OMIM 225400) is a rare inheritable connective tissue disorder characterized by a deficiency of collagen lysyl hydroxylase 1 (LH1; EC 1.14.11.4) due to mutations in *PLOD1*. Biochemically this results in underhydroxylation of collagen lysyl residues and, hence, an abnormal pattern of lysyl pyridinoline (LP) and hydroxylysyl pyridinoline (HP) crosslinks excreted in the urine. Clinically the disorder is characterized by hypotonia and kyphoscoliosis at birth, joint hypermobility, and skin hyperelasticity and fragility. Severe hypotonia usually leads to delay in gross motor development, whereas cognitive development is reported to be normal.

**Methods:**

We describe the clinical, biochemical and molecular characterisation, as well as electron microscopy findings of skin, in 15 patients newly diagnosed with this rare type of Ehlers-Danlos syndrome.

**Results:**

Age at diagnosis ranged from 5 months to 27 years, with only 1/3 of the patients been diagnosed correctly in the first year of life. A similar disease frequency was found in females and males, however a broad disease severity spectrum (intra- and interfamilial), independent of molecular background or biochemical phenotype, was observed. Kyphoscoliosis, one of the main clinical features was not present at birth in 4 patients. Importantly we also noted the occurrence of vascular rupture antenatally and postnatally, as well as developmental delay in 5 patients.

**Conclusion:**

In view of these findings we propose that EDS VIA is a highly variable clinical entity, presenting with a broad clinical spectrum, which may also be associated with cognitive delay and an increased risk for vascular events. Genotype/phenotype association studies and additional molecular investigations in more extended EDS VIA populations will be necessary to further elucidate the cause of the variability of the disease severity.

## Background

The kyphoscoliotic type of Ehlers-Danlos syndrome (EDS), type VIA (MIM 225400) is a rare autosomal recessively inherited connective tissue disorder with a disease incidence of approximately 1;100,000 live births. The disorder is characterized at birth by severe muscular hypotonia often requiring invasive neuromuscular work-up, kyphoscoliosis which is progressive and severe, marked joint hypermobility and luxations, and severe skin hyperelasticity. In addition, there is fragility of the skin with abnormal scarring, osteopenia without tendency to fractures, often a striking Marfanoid habitus and microcornea, bluish sclera, and occasionally rupture of arteries and the eye globe, in contrast to rupture of the cornea reported in brittle cornea syndrome [1-3]. Intellect is reportedly unaffected.

The kyphoscoliotic type of EDS was the first inborn error of human collagen metabolism to be defined at the biochemical level as early as 1972, based on a family study in which two sisters had severe progressive scoliosis since early infancy, marked joint laxity and recurrent joint dislocations [4,5]. It is caused by a deficiency of the collagen-modifying enzyme procollagen-lysine, 2-oxoglutarate 5-dioxygenase 1 (LH1; EC 1.14.11.4; MIM 153454; PLOD1 or lysyl hydroxylase 1) [4] due to homozygosity or compound heterozygosity for a mutant *PLOD1 *allele(s) [1,6]

This enzyme plays an important role as a post-translational modifying enzyme in collagen biosynthesis through both hydroxylation of lysyl residues in -Xaa-Lys-Gly- collagen sequences to hydroxylysyl residues which serve as sites of attachment for carbohydrate units (either galactose or glucosyl-galactose) and in the formation of intra- and intermolecular collagen cross-links [7]. Thus, lysyl hydroxylase deficiency results in underhydroxylation of lysyl residues and underglycosylation of hydroxylysyl residues in collagens and, hence, impaired cross-link formation with consequent mechanical instability of the affected tissues. However, the underlying pathogenetic mechanism is not yet fully understood.

As a result of underhydroxylation and underglycosylation, the collagen α-chains display a faster electrophoretic mobility on sodium dodecyl sulfate polyacrylamide gel electrophoresis (SDS-PAGE), which in turn is used as a diagnostic test [8].

Furthermore, the enzyme deficiency gives rise to an abnormal urinary excretion pattern of lysyl-pyridinolines (LP) and hydroxylysyl-pyridinolines (HP) cross-links. The ratio of urinary total LP to HP in patients with EDS type VIA is high as compared with normal controls [9] and is diagnostic for EDS type VIA [6,10,11]. The diagnosis of EDS type VIA may then be confirmed by measuring the activity of the enzyme in cultured skin fibroblasts [12] and/or directly by mutation analysis of *PLOD1 *(MIM 153454). More than 30 different mutations in *PLOD1 *have been described in the kyhposcoliotic form of EDS [13-15]; among them an 8.9-kb duplication of seven exons (exon 10-16) is the most common cause of the disorder.

The kyphoscoliotic type of EDS has some overlapping clinical features with other forms of EDS as well as with a new recessive entity, with fleshy swelling of facial tissues, severe scoliosis and ocular fragility, *RIN2 *syndrome [16]. Diagnosis is usually delayed because a neuromuscular disease is suspected due to the severe neonatal hypotonia and delay in gross motor development. It is only after negative neuromuscular workup that in most cases EDS VIA is suspected.

Intra- and interfamiliar variability of clinical severity has been observed in individuals with the kyphoscoliotic form of EDS [2,13], however no relationship between position or type of mutation in the *PLOD1 *gene and the severity of the clinical phenotype was observed so far.

Over a period of 1.5 years, 12 new index patients and 3 relatives were diagnosed with this relatively rare type of Ehlers Danlos syndrome at our Institution. Here we present results on individual clinical presentation, and highlight clinical features that are striking for the diagnosis of EDS VIA, but also focus on rare and therefore often underestimated complications including artery rupture and developmental delay. In addition, we correlate the clinical picture with biochemical and molecular results. Thus this paper gives an overview on clinical symptoms, describes the broad phenotypic spectrum of the kyphoscoliotic type of EDS, and highlights the specificity of non invasive urinary analysis of pyridinolines which is a fast and cost effective diagnostic test for the kyphoscoliotic form of EDS.

## Methods and Materials

### Clinical information

Clinical information was obtained through a questionnaire that was sent to each involved centre and subsequently analysed centrally. Clinical examination was in most cases performed by a geneticist or a paediatrician. Diagnosis of EDS VIA was based on clinical grounds in all index patients and proven by the abnormal urinary ratios of total pyridinolines, as shown to be diagnostic for EDS VIA [2,10,11]. Mutation analysis of *PLOD1 *was done in all but two (P8 and P11) patients, for whom DNA was not available. For all studies informed consent of the patients or their parents, in accordance with requirements of the Local Ethics Committees of the referring physicians was obtained.

### Biochemical analyses and electron microscopy

Total urinary pyridinolines were measured as described [9,11] and were expressed as the ratio of lysyl pyridinoline (LP) to hydroxylysyl pyridinoline (HP).

Dermal fibroblast cultures were established from skin biopsies from 4 patients (P1, P2, P7a, P10) by routine procedures. Cells were maintained under standard conditions, and radiolabelled collagen samples were prepared by digestion with pepsin, precipitated with ethanol, separated on a 5% SDS- polyacrylamide gel, and visualized by fluorography [17]. For electron microscopy skin biopsies were processed for transmission electron microscopy as reported [18].

### Mutation analysis

gDNA was isolated from peripheral blood leukocytes or cultured fibroblasts using standard techniques. Each single exon of *PLOD1 *was amplified by PCR using 5' and 3' primers designed on the flanking intron sequences as described [13].

Mutation analysis by sequencing was performed using the Big-Dye Terminator cycle sequencing ready-reaction kit version 1.1 (Life Technologies, Applied Biosystems) and an AB 3130 Genetic Analyzer (Life Technologies, Applied Biosystems), as described [13].

## Results

### Clinical features

The available clinical data of all index patients and relatives (siblings/cousin) are summarized in Table 1. All index patients, suspected with EDS VIA based on clinical findings, were referred to our centre for biochemical workup of EDS VIA, respectively urinary pyridinolines as well as molecular analysis. Mean age at diagnosis was 6.3 years (range 5 month - 27 years), with 53% male and 47% female patients. 2/3 of all index patients were diagnosed before 5 years of age, 4 out of 8 patients in the first year of life. One patient was diagnosed in adulthood with no clinical suspicion for a connective tissue disorder. Most patients were reported to be a product of consanguineous parents; 75% of the patients originating from Turkey, former Yugoslavia and the Middle East, while the remaining originated from Western Europe and Somalia.

**Table 1:** Clinical, molecular and biochemical characterization of 15 new EDS VIA patients.

	P1	P2	P3	P4a	P4b	P5	P6	P7a	P7b	P7c	P8	P9	P10	P11	P12
Sex	M	M	F	M	M	M	F	F	F	M	M	M	F	F	F

Age at diagnosis	9 m	27 y	14 y	4 y	6 m	9 y	5 m	15 m	14 m	13 m	19 m	16 y	16 y	18 m	8 m

Ethnicity	Mazedonia	Serbia	Iran	Turkey	Turkey	Turkey	Netherland	Somalia	Somalia	Somalia	Turkey	Turkey	Spain	Turkey	Iraqui

Consanguinity	+	-	+	+	+	+	-	+	+	+	+	+	+	+	+

Urinary LP/HP #	8.16	5.47	5.59	4.02	9.5	5.15	8.48	8.32	9.03	not done	6.98	6.1	6.51	6.6	7.8

*PLOD1 *mutation	Dup/Dup	p.L85P/p.L85P *	c.1471-1 G > A/ c.1471-1 G > A*	Dup/Dup	Dup/Dup	c.1095C > T/ c.1095C > T*	c.1651-2 A > G/ c.1651-2 A > G*	p.G678R/p.G678R	p.G678R/ p.G678R	p.G678R/ p.G678R	*DNA not available*	p.Q345X/ p.Q345X*	c.466+ 1G > A/ c.466+ 1G > A*	*DNA not available*	p.Trp419leufsX48*/ p.Trp419leufsX48*

muscular hypotonia at birth	+	-	+	+	+	+	+	+	+	+	+	+	+	+	+

Kyphoscoliosis at birth	+	-	+	+	+	+	+	+	-	-	+	+	-	+	+

Marfanoid habitus	+	+	+	+	+	+	+	+	+	-	-	+	-	-	+

Delayed gross motor development	+	-	+	+	+	+	+	+	+	+	+	+	+	+	+

delayed cognitive development	-	-	-	-	-	-	-	-	-	-	+	+	+	+	+

Rupture of artery	-	+	-	-	-	-	-	-	-	-	-	-	-	-	+

Smooth velvety skin	+	+	+	+	+	+	+	+	+	+	+	+	+	+	+

**Extensibel fragile skin**	**-**	**-**	**+**	**?**	**?**	**+**	**+**	**?**	**+**	**+**	**+**	**?**	**+**	**?**	**+**

**Joint laxity**	**+**	**+**	**+**	**+**	**+**	**+**	**?**	**+**	**+**	**+**	**+**	**+**	**+**	**+**	**+**

Hip dislocation at birth	+	-	+	-	-	+	-	-	-	-	+	-	-	-	+

Neuromuscular workup	+	-	-	-	-	+	-	+	-	-	+	-	+	+	-

Independent walking at age	2 y	1.5 y	4 y	?	?	?	2 y	n.a.	°	2 y	n.a	n.a	2 y	°	°

															

*newly described mutation															

+, Finding present; -, finding absent; ? no information available,; °, patient < 24 months at last exam; n.a, not able to; m, month and y, year and ^# ^LP/HP in normal control: 0.20 ± 0.05 (range 0.10-0.38); *, newly described mutation; ^§^, common duplication of exon 10 to exon 16.

Except for P2, who had a surprisingly mild phenotype, the patients were all noted to have with severe neonatal muscular hypotonia and were described as floppy babies, with a poor cry and difficulty in sucking. A neuromuscular disorder was suspected with subsequent invasive muscular work-up in 7 patients (P1, P2, P5, P7a, P8, P10, P11), including CK, electromyography, ultrasound of muscle, and brain computer tomography or magnetic resonance imaging (MRI); all results were normal. Kyphoscoliosis was one of the main features presenting at birth and was severe and progressive in up to 80%. P10 developed kyphoscoliosis only at the age of 10 years and P7c at the age of 20 months. All patients presented with joint hypermobility, and some had luxations of the hip joints at birth or later in life (P1, P3, P5, P8, and P12). The skin was smooth and hyperelastic in all, however easy bruising with poor wound healing and atrophic scars were noted only in P2 and P3.

Antenatal/neonatal brain haemorrhages was documented in P12 and rupture of coronary arteries in one patient (P2). 4/12 patients diagnosed with EDSVIA with negative neurological workup had delay of cognitive development.

### Detailed history of selected patients

#### P2

A 27 year old man was the 2nd child of apparently non-consanguineous parents, which were born and raised within 5 km distance. There is only limited information available about his childhood; however the patient's father did not recall any significant differences regarding the motor and cognitive milestones. His skin was reported to be smooth with history of easy bruising. The patient complained about abnormal, delayed wound healing resulting in hypertrophic scars. His general health however was normal, and he was full time working as a constructor. It was at the age of 27 years when he and a co-worker both presented at the emergency ward with nausea, dyspnoe and retrosternal pain. CO_2 _intoxication was suspected. After 24 hours hospitalisation he deteriorated rapidly with tachycardia and ejection fraction of only 50%. Subsequently a coronary angiography was performed to elucidate the cause of his cardiopathy. During the coronary angiography he had spontaneous dissection of the ramus interventricularis anterior (RIVA) and main coronary artery causing acute cardiac failure. The patient's older brother died at the age of 20 years after an accidental fall from a balcony (1.5 m height); he apparently suffered from arterial bleedings that could not be managed.

#### P6

She is the first child of healthy Dutch parents with no known consanguinity. She was born after an uneventful pregnancy at 41 weeks of gestation by caesarean section because of breech presentation. Mild oligohydramnios was noted. Birthweight was 3.7 kg and length 55 cm (> P97). At birth severe hypotonia with an abnormal position of hands and contractures of both feet, as well as velvety skin were noted. There was a mild kyphoscoliosis and hypermobility of the joints. At 5 months of age she presented with significant motor delay, which improved with physiotherapy. At the same time she presented with axial and peripheral hypotonia as well as severe hypermobility of the joints. Growth was at the 97^th ^percentile. She showed mild dysmorphic features with hypotelorism, narrow palpebral fissures and an open mouth. Her palate was high and narrow. She had kyphoscoliosis and a pectus excavatum. At age 23 months she was able to stand unsupported and walk with, but not without, support. Mental development and speech development was normal.

#### P7a-7c

Patient 7a is the first child of consanguineous Somali parents. Antenatally there were concerns about poor fetal growth, along with oligohydramnios and reduced fetal movements. She was born at 38 weeks of gestation; birthweight was 2.8 kg (25^th ^percentile). She was noted with muscular hypotonia and contractures of the hands and feet. When evaluated by paediatric neurologists, history of delayed gross motor milestones was noted and a possible diagnosis of a congenital myopathy was considered. Open muscle biopsy (left thigh) showed variable fibre size with mild fibrosis. Immunohistochemistry for dystrophin, merosin, sarcoglycan and collagen VI were reported as normal, with normal respiratory chain enzyme studies. Assessment in the genetic clinic at 16 months of age noted that the patient was unable to walk independently, and required a spinal brace and leg splints. Poor weight gain was reported in infancy due to gastro-oesophageal reflux. There were no concerns about cognitive development, hearing or vision. There was marked joint laxity in all joints with soft skin texture and blue sclera. She had severe and progressive scoliosis; placement of posterior spinal rods was necessary at the age of 3 years. Due to an infectious complications rods had to be removed, subsequently her scoliosis was measured > 100 degrees. There were no known cardiac complications.

Patient 7b was born at 38 weeks of gestation with a birth weight of 2.5 kg (9^th^-25^th ^percentiles). There were no abnormal findings antenatally. At 6 weeks of age she was noted to be hypotonic with joint laxity, long and thin fingers, soft skin and subcutaneous bruising. Ophthalmological review at 6 months of age showed normal visual acuity, with no evidence of myopia. There were no known cardiac complications.

Patient 7c is the fifth child and was born at term with a birth weight of 3.1 kg (25^th ^percentile). She is the cousin of P7a and P7b. She was noted with muscular hypotonia at birth; karyotype, baseline metabolic investigations and MRI brain scan were reported to be normal. Paediatric neurology review noted gross motor delay with no evidence of visual, auditory or cognitive impairment. Connective tissue abnormalities included generalised joint laxity, soft hyperextensible skin and long and thin fingers. Cardiac review and echocardiogram were normal. Ophthalmology noted bilateral myopia (-3 Dioptres) with no structural abnormalities. Orthopaedic review at 23 months noted a slight kyphosis not regarded as requiring any intervention.

#### P8

She is the first child, born at term after uneventful pregnancy to consanguineous (first degree cousins) parents. Her weight and height were at 10^th ^percentile, while her head circumference was at 50^th ^percentile. She was noted at birth with severe muscular hypotonia and kyphoscoliosis as well as smooth, hyperelastic and fragile skin. On both hands and feet she presented flexible fingers and toes and joint hypermobility. She had some dysmorphic features such as low-set ears, broad nasal bridge, frontal bossing, short philtrum and short neck. At the age of 2 years she presented with motor and metal developmental delay, and was not yet able to walk independently. Brain MRI was normal.

#### P9

This is the second child to 1.5 degrees cousins, born at term after an uneventful pregnancy. His birth weight was 2.8 kg, height was not recorded. At the age of 6 months, hydrocephalus was detected and subsequently treated with craniocervical surgery. Already at that point of time he showed delayed developmental milestones, but it was only at the age of 13 years that he was referred for evaluation of mental and motor delay as well as skeletal deformity. He has never walked. Physical examination revealed that his percentiles were within the normal range. He was noted to have strabismus, asymmetric face, minimal prognathism, pectus excavatum, scoliosis, 2nd-3rd toes syndactyly on feet bilaterally, generalized hypotonia, joint laxity, and mental retardation. Skeletal X-ray showed severe scoliosis and hip dysplasia. Cranial MRI showed mild hydrocephalus (operated). Karyotype was normal. Family history revealed a sibling with similar clinical findings who died at the age of 17 years from complications of a sudden internal hemorrhage (DD: aortic aneurysm). He had severe skeletal deformities including kyphosis and joint laxity and soft skin; and developmental delay. There was no autopsy performed.

#### P10

She is the first of four children born to healthy consanguineous parents (uncle-niece union) after an uneventful pregnancy. Delivery was normal. No kyphoscoliosis or deformities were noted at birth. In the neonatal period she was noted to be slightly hypotonic, however her growth and development were normal in the first year of life. At the age of 2 years a speech delay was first noted, and subsequently a more global delay became apparent at the Nursery. An extended neurological workup including metabolic screen, chromosomes, and brain CT did not reveal an underlying cause for her delay. Physical examination was unremarkable. At the age of 6 years she developed epileptic seizures; brain MRI showed non-specific white matter changes. She was started on valproic acid with good response to treatment. The epileptic activity on initial EEG could not be confirmed on follow up EEG. Scoliosis was first noted at the age of 10 years with little progression over the following years. She has mild-moderate delay and attends a Special Needs School. Due to her body habitus Marfan syndrome was recently suspected. Echocardiography showed a mild mitral regurgitation with a normal aortic root diameter. She showed marked joint laxity (Beighton score: 8/9), skin hyperelasticity with smooth velvety and doughy feel to it, and atrophic scars on both knees and back. She had a mild chest deformity with asymmetry and pectus carinatum and moderate scoliosis with slender arms and legs accompanied by arachnodactyly. Her facial features were slightly dysmorphic. She had a high arched palate and dental crowding. Repeat MRI at the age of 16 years showed subtle and non-specific linear white matter changes on the right parietal region, with no evidence of heterotopias or any other structural anomaly.

#### P12

This is the third child of consanguineous (double first cousin) parents. The pregnancy was complicated with gestational diabetes and detection of hydrocephalus at 34 weeks of gestation. There was spontaneous onset of labour at 38 weeks and she was born with Apgar scores of 9 and 9 at 1 and 5 minutes, respectively, and a birth weight of 2.8 kg (25th percentile). Congenital kyphosis was noted. Postnatal cerebral MRI confirmed hydrocephalus which was presumed to be secondary to multiple extensive intraventicular and intraparenchymal haemorrhages. Thrombotic screen was normal. Since then there has been a history of developmental delay, marked hypotonia, and failure to thrive. At 4.5 month of age her weight was 4.5 kg (< 3rd percentile), her height was 60 cm (25 percentile) and the head circumference was 38 cm (< 3rd percentile). There was minimal subcutaneous tissue and fat stores. Dolicocephaly, a prominent metopic suture and a ridged sagittal suture, and a myopathic facies with retrognathia were noted, as well as a left convergent strabismus, telecanthus and epicanthic eye folds. Her palate was very high and narrow. She had a marked kyphosis, arachnodactyly with hypermobile digits and soft smooth skin. There was peripheral hypotonia, with a striking lack of spontaneous hand movement and her wrists were held in a 'wrist drop' position. Knee jerks were increased and plantar responses were down going. She did smile and make eye contact with her mother at age 4.5 months but there was little developmental progress at the follow up visit at age 8.5 months. Transferrin isoforms, repeat CK, karyotype and chromosomal microarray were normal. Nerve conduction studies were also normal.

### Biochemical analyses

The urinary ratios of total pyridinolines (LP to HP) were markedly increased in all patients (mean 7.03 range 4.0 - 9.5), as compared to those of control samples (0.20 ± 0.05, range 0.10-0.38). These findings were consistent with the diagnosis of Ehlers-Danlos syndrome, kyphoscoliotic type (EDS VI; LP/HP 5.99 ± 0.99; range 4.30-8.10; n = 17). LP to HP ratio was significantly higher in the group 0-4 years, compared to the age group of 4 years and older (p = 0.02)

Collagen biochemical analysis was performed in P1, P2, P7a and P10, only. The electrophoretic migration of the α-chains of collagen types I, III, and V and their precursors and cross-linked products synthesized by cultured dermal fibroblasts, was increased in all patients upon SDS-PAGE (see Figure 6 in Jarisch et al., 1998, for a similar example), and demonstrates indirectly that lysyl residues are underhydroxylated and underglycosylated [2].

**Figure 1:**
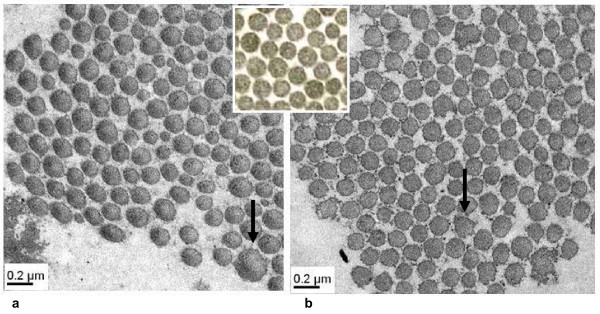
**Electron microscopic findings of the skin of patient P1 (a) and P2 (b) compared with normal control (middle)**. Collagen fibrils are of variable diameters and are irregularly spaced. The contour is variable from round in small fibrils to jagged in large fibrils (arrow).

Electron microscopy of skin was performed on patients 1 and 2 and showed identical abnormalities (Figure 1) including, variable diameters and abnormal contours of the collagen fibrils when compared to normal controls. Diameters of the collagen fibrils were significantly increased with loss of the normal circular shape. In particular those fibrils with abnormally increased diameters showed a greater deviation from circular shape (Figure 1). The elastic fibers presented normal ultrastructural features.

### Molecular Analysis

Results of mutation analysis of *PLOD1 *performed on genomic DNA of the patients [13] are reported in Table 1.

13 patients had molecular testing of *PLOD1*. All were confirmed to carry homozygous mutations (Table 1). In 3/13 the 8.9-kb duplication of seven exons (exon 10-16) was identified. In 7 patients previously unreported mutations were found including 4 mutations expected to affect splicing. Due to the unavailability of RNA, the mutations localized at 5' and 3' splice donor and acceptor sites, respectively, were analysed in *silico *for the prediction of aberrant splicing using NetGene2 Server available at http://www.cbs.dtu.dk/services/NetGene2[19]; furthermore, the effects of missense mutations on aberrant splicing was evaluated in silico using the ESEfinder software available at http://rulai.cshl.edu/cgi-bin/tools/ESE3/esefinder.cgi[20,21]. 1) The c.466+1G > A at the donor splice site of exon 4, is predicted to cause skipping of exon 4. 2) The c.1095C > T transition in exon 10 is expected to activate a cryptic splice site within exon 10 which would promote aberrant splicing, shift of the open reading frame and premature stop at codon 386. 3) The G > A substitution at position -1 of the 3' acceptor splice site of exon 14 (c.1471-1 G > A) is expected to cause the activation of a cryptic splice site within exon 14, causing an out of frame deletion of the first 55 bp of exon 14 leading to a premature stop in exon 17. 4) The c.1651-2 A > G mutation at the 3' acceptor site of exon 16, is expected to affect splicing of exon 16, leading to skipping of exon 16.

## Discussion

The kyphoscoliotic type of the Ehlers-Danlos syndrome (EDS type VIA) is a rare autosomal recessive connective tissue disorder, which in the first years of life mainly affects the musculoskeletal system. Infants affected with EDS type VIA present with moderate to severe generalized muscle hypotonia, and with skeletal findings such as kyphoscoliosis and joint hyperextensibility which are also common to neuromuscular disorders such as congenital myopathies including Bethlem myopathy and Ullrich muscular dystrophy [22] as well as lower motor neuron diseases, especially when deformities of the feet and joint dislocations coexist. In all patients documented in the literature [2,11] kyphoscoliosis is usually present at birth, and hypotonia in conjunction with the inability to stabilize the joints, due to laxity of the connective tissue, leads to a delay in gross motor development in the first years of life. The kyphoscoliotic form of EDS can be diagnosed by non invasive biochemical analysis of urinary pyridinolines; molecular analysis of the *PLOD1 *gene can be added for future prenatal diagnosis. Variability of the clinical phenotype is suspected even though limited data is available about the spectrum of the disease severity; in particular little data is available about the presentation in adulthood. Thus, here we report on the broad clinical spectrum found in 12 index patient and 3 relatives diagnosed between the age of 7 months and 27 years in order to emphasize the clinical features that add insight into the clinical variability of the disease. Furthermore, we focus on biochemical and molecular aspects, as well as electron microscopy findings of relevance for a timely diagnosis.

The newborns with available history and biochemically confirmed EDS type VIA are generally described with hypotonia and delay of motor development in the first few years of life [22]. All but one index case (P2) presented with significant muscular hypotonia that was noted at birth. This patient was first diagnosed at the age of 27 years and his family was not aware of any problems within his first few years of life. We can not exclude however, that this clinical feature was present but further ignored, as it is generally believed that motor development and muscular hypotonia presenting in the neonatal period improve over the course of time even though kyphoscoliosis is usually progressive.

Currently, very little is known about long-term outcome of ambulation in patients with EDS VIA, therefore, we present here for the first time data about the ability of walking in a collective of 15 patients. Notably, one patient was wheel chair bound and never able to walk and two were reported to walk with assistance only. It might be speculated that the absence of neonatal kyphoscoliosis is a positive prognostic factor regarding future ambulation, since all our patients without kyphoscoliosis at birth were able to walk. However, in the light of the small number of affected individuals this assumption might be taken with caution. Based on the results of 3 related individuals from consanguineous parents who shared the mutant *PLOD1 *genotype, but did not develop the same severity and age of onset of kyphoscoliosis, we conclude that this clinical feature might not correlate with the genotype.

A feature that was present in all patients was smooth velvety skin; it should therefore be included to the specific diseases features/characteristics. In particular, velvety skin texture may help to distinguish EDS, kyphoscolitic form from congenital myopathies.

Kyphoscoliosisis, attributed to muscular hypotonia together with ligamentous laxity, is generally also reported at birth and represents one of the striking hallmarks of this disease; it should prompt clinician to the diagnosis of EDS type VIA as it is usually not found in neuromuscular diseases. Surprisingly, kyphoscoliosis at birth was not reported in 4 of our patients.

However, two of them developed kyphoscoliosis later in life (Table 1), while P2 did not develop any asymmetry of the spine until the age of 27 years. Interestingly, this patient did not present with hypotonia in childhood, in contrast to the other two, suggesting that lack of hypotonia at birth might be a favorable prognostic finding for non developing spine deformity later in life. Indeed, data from *PLOD1^-/- ^*mice suggest a direct correlation between lack of kyphosis and absence of muscle hypotonia at birth [23].

In addition, no association between the presence of kyphoscoliosis at birth and the type of *PLOD1 *mutation was found in our collective of EDS VIA.

A lack of association between genotype and phenotype has recently been suspected [2,13] and our findings reflect again the phenomenon of interfamilial and intrafamilial variability of this disorder. The list of disorders where the same mutation presents with a different phenotype is steadily increasing and includes, for example, mutations of *COLA1 *in osteogenesis imperfecta. Modifying sequence variants within the entire genome, epigenetic factors or environmental factors may be responsible for this observation.

In contrast to other EDS forms such as EDS type VII [24], hip dislocations at birth seems to be a less common feature, described in only 25% of our patients.

Importantly and adding to the current knowledge of EDS VIA, we noted the occurrence of vascular rupture both, antenatally in one patient (P12) as well as in adulthood as the first clinical symptom (P2). Vascular rupture is by far the major life-threatening complication in this disorder however it is thought to be less frequent than in EDS type IV. Antenatal vascular event was previously reported in two patients with EDS VIA, only [13]. The finding of antenatal vascular events in our EDS VIA collective increases the likelihood of their direct association with EDS VIA. Furthermore, vascular events might be more frequent in EDS VIA than expected. Notably, 15% of *PLOD1^-/- ^*mice died at the age of 1-4 months due to aortic dissections, caused most likely by degenerated smooth muscle cells and abnormal collagen fibrils of aortic wall [23]. Thus antenatal vascular events of unknown aetiology should therefore prompt investigations for EDS type VIA, Historically, intellect is reported to be unaffected in kyphoscoliotic type of EDS. Unexpectedly, we found delayed cognitive function in 5 out of 12 index patients, however, except for the patient with antenatal central nervous system vascular event, brain imaging and neurological exam did not reveal an underlying cause for the developmental delay. Whether the impaired cognition is in direct association with EDS VIA or independent and therefore not part of the disease remains to be further investigated. Further, in the context of consanguinity a concomitant disorder must be taken into consideration.

Kyphoscoliotic type of EDS is diagnosed by abnormally elevated ratio of urinary lysyl pyridinolines to hydroxylysyl pyridinoline crosslinks and can be confirmed by mutation analysis of *PLOD1*. Our results on LP/HP ratios indicate that the ratio is highest in the age group 0-4 year and subsequently decreases, however remains significantly elevated when compared to normal controls. No correlation between LP/HP ratios and genotype were observed. In all but 2 patients molecular analysis of *PLOD1 *was performed confirming the diagnosis based on pyridinoline ratios in urine (Table 1).

Specific abnormal collagen electron microscopy findings in skin biopsies have been described for classical EDS [18], EDS VII [25] and recently also for *RIN2*-deficient patients [16]. Here we describe typical collagen fibril alterations in skin biopsies of two EDS VIA patients. Abnormal electron microscopy findings (Figure 1) included variable diameters of the collagen fibrils, irregular interfibrillar space in addition to irregular ragged outlines in cross sections. To our knowledge these findings are distinct and have not been described in other subtypes of EDS or other connective tissue disorders. Furthermore, they resemble those described in *PLOD1^-/- ^*mice [23].

In summary we present 15 additional individuals with kyphoscoliotic type of EDS. In all but one (sibling of an index case), the diagnosis was made on clinical grounds and subsequently confirmed by analysis of pyridinolines in urine, as well as molecular studies of *PLOD1*. Determination of urinary pyridinoline cross-links is a fast, non-invasive, simple, and indeed reliable diagnostic test for EDS type VI.

Typical clinical features included muscular hypotonia at birth and smooth velvety skin. However adding to the current knowledge, kyphoscoliosis was not necessary present at birth/diagnosis but was reported to develop later in life in some individuals. In addition, kyphoscoliosis represents a clinical feature of EDS type VIA with a high frequency of inter- and intrafamilial variability regarding severity and age of onset with no obvious association to the *PLOD1 *gene genotype. Age of onset of kyphoscoliosis might indirectly be of importance for progress or delay of motor developmental milestones and thus might reflect a prognostic factor for achievement of ambulation. In addition, but of utmost importance a less severe clinical phenotype seems to exist, only presenting in adulthood with acute life threatening vascular events and velvety skin, without skeletal involvement. Clinical data of additional patients is required to validate our findings and to investigate possible factors contributing to inter- and intrafamilial variability.

## Conclusion

In conclusion EDS VIA, the kyphoscoliotic type of EDS is a rare disease which should be considered in any individual presenting with muscular hypotonia, progressive kyphoscoliosis or antenatal vascular event of unknown origin. Determination of urinary pyridinoline cross-links is a fast, non-invasive, simple, and indeed reliable diagnostic test, which might improve diagnosis of a yet under diagnosed disease.

## Competing interests

The authors declare that they have no competing interests.

## Authors' contributions

MR: conception of the study, coordination and collection of all data, writing the drafts; AV: referred 3 patients, wrote detailed patient history, helped to draft the manuscript; UY: referred 3 patients, wrote detailed patient history, helped to draft the manuscript; GS: referred 2 patients, wrote detailed patient history, helped to draft the manuscript; EA: referred 1 patients, wrote detailed patient history, helped to draft the manuscript; MC and KJ: referred 1 patients, wrote detailed patient history, helped to draft the manuscript; SG: referred 1 patients, wrote detailed patient history, helped to draft the manuscript; AK: referred 1 patients, wrote detailed patient history, helped to draft the manuscript; MK: carried our measurement of urine pyridinolines; CM: referred 1 patients, wrote detailed patient history, helped to draft the manuscript; MB: helped to draft the manuscript and revised the manuscript; CG: conception of the study, carried out molecular genetic studies, as well as biochemical analysis of fibroblasts, helped to draft the manuscript.

All authors read and approved the final manuscript.
